# Binding of Hepatitis B Virus Pre-S1 Domain-Derived Synthetic Myristoylated Peptide to Scavenger Receptor Class B Type 1 with Differential Properties from Sodium Taurocholate Cotransporting Polypeptide

**DOI:** 10.3390/v14010105

**Published:** 2022-01-07

**Authors:** Shuji Hinuma, Shun’ichi Kuroda

**Affiliations:** The Institute of Scientific and Industrial Research (SANKEN), Osaka University, Mihogaoka 8-1, Ibaraki 567-0047, Osaka , Japan

**Keywords:** hepatitis B virus (HBV), myristoylated pre-S1 peptide (Myr47), scavenger receptor class B type 1 (SR-B1), sodium taurocholate cotransporting polypeptide (NTCP), HEK293T cells, yeast-derived nanoparticles containing L protein (bio-nanocapsules: BNCs), endocytosis, crosslinker, pull-down assays

## Abstract

(1) Background: The myristoylated pre-S1 peptide (Myr47) synthesized to mimic pre-S1 domain (2-48) in large (L) surface protein of hepatitis B virus (HBV) prevents HBV infection to hepatocytes by binding to sodium taurocholate cotransporting polypeptide (NTCP). We previously demonstrated that yeast-derived nanoparticles containing L protein (bio-nanocapsules: BNCs) bind scavenger receptor class B type 1 (SR-B1). In this study, we examined the binding of Mry47 to SR-B1. (2) Methods: The binding and endocytosis of fluorescence-labeled Myr47 to SR-B1 (and its mutants)-green fluorescence protein (GFP) fusion proteins expressed in HEK293T cells were analyzed using flow cytometry and laser scanning microscopy (LSM). Various ligand-binding properties were compared between SR-B1-GFP and NTCP-GFP. Furthermore, the binding of biotinylated Myr47 to SR-B1-GFP expressed on HEK293T cells was analyzed via pull-down assays using a crosslinker and streptavidin-conjugated beads. (3) Conclusions: SR-B1 bound not only Myr47 but also its myristoylated analog and BNCs, but failed to bind a peptide without myristoylation. However, NTCP only bound Myr47 among the ligands tested. Studies using SR-B1 mutants suggested that both BNCs and Myr47 bind to similar sites of SR-B1. Crosslinking studies indicated that Myr47 binds preferentially SR-B1 multimer than monomer in both HEK293T and HepG2 cells.

## 1. Introduction

The HBV genome encodes surface proteins that are large (L protein: pre-S1 + pre-S2 + S regions), middle (M protein: pre-S2 + S regions), and small (S protein: S region) [1]. In these surface proteins, the pre-S1 domain of L protein plays a crucial role in the infection of HBV [2,3]. In addition, a synthetic myristoylated pre-S1 peptide (i.e., Myr47) has been reported to suppress HBV infection [4,5,6,7]. It has been proven that Myr47 binds NTCP (encoded by the *SLC10A1* gene), and its binding inhibits HBV infection to hepatocytes [8]. The N-terminal myristoylation of pre-S1 is essential to bind NTCP and to prevent HBV infection [4,5,6,7,8]. As to the entry process of HBV/hepatitis D virus (HDV) into host cells, it is currently presumed that viral particles bind heparan sulfate proteoglycans (HSPG) and NTCP on the surface of hepatic cells, and then a complex of HBV and receptors are endocytosed; their genome is finally released into the cytoplasm of host cells, although the precise mechanism of this process is still unclear [9,10]. Myr47 (also referred to as bulevirtide) is used to treat patients infected with HDV/HBV [11,12]. On the other hand, BNCs with myristoylated N-terminal L protein on their surfaces are endocytosed in a hepatic cell line, HepG2, independent of NTCP expression [10]. Therefore, there is a possibility that myristoylated L protein or Myr47 can bind other receptors different from NTCP. Furthermore, it is suggested that Myr47 interacts with phospholipid membranes in a nonspecific manner [13]. However, little is known about a definitive receptor which can bind Myr47 other than NTCP.

Our studies on the interaction between phospholipid nanoparticles (PNPs) (e.g., liposomes and exosomes) and cells revealed that there is a common response to PNPs among various mammalian cells. PNPs are endocytosed and induce lipid droplet (LD) formation in the cytoplasm of most mammalian cells [14]. During the exploitation of a mechanism for this cellular response, we found that liposomes consisting of phosphatidylethanolamine (PE) and phosphatidylcholine (PC) are efficiently incorporated into HEK293T cells via SR-B1 (encoded by the *SCARB1* gene) [15]. We also demonstrated that liposomes containing PC bind SR-B1, and this binding can be modulated by phosphatidic acid [16]. Although SR-B1 is the primary receptor for high-density lipoprotein (HDL), it has been reported to bind a variety of ligands, including liposomes, composed of phosphatidylserine (PS) other than HDL [17]. A series of our studies demonstrated that SR-B1 can function for cells to recognize various PNPs and to endocytose them.

As yeast-derived nanoparticles coated with recombinant L protein have been expected to be useful as a carrier to deliver bioactive substances for hepatocytes and the liver, they are sometimes referred to as BNCs [18,19,20]. However, BNCs are not myristoylated and they cannot bind NTCP. Although HSPG was assumed to be a candidate receptor for BNCs, it was ambiguous as to which receptor contributes to the cellular binding of BNCs [10]. As BNCs can be regarded as a sort of PNPs, we examined the binding of BNCs to SR-B1 and found that they can bind SR-B1 in HEK293T cells [21]. BNCs and Myr47 share commonalities because both are composed of a part of HBV structural components. As BNCs bind SR-B1, we examined whether Myr47 can interact with SR-B1 too. In this study, we report the unique binding properties of SR-B1 to Myr47.

## 2. Materials and Methods

### 2.1. Myr47 and Its Derivatives

Biotinylated Myr47 (Myr-GTNLSVPNPLGFFPDHQLDPAFGANSNNPDWDFNPNKDQWPEANQVK-biotin) was purchased from Scrum, Inc., Tokyo, Japan. Biotinylated Myr47 derivatives, D-11/13 (Myr-GTNLSVPNpLgFFPDHQLDPAFGANSNNPDWDFNPNKDQWPEANQVK-biotin, in which d-amino acids were indicated in lower-case letters) and aa2–48 (GTNLSVPNPLGFFPDHQLDPAFGANSNNPDWDFNPNKDQWPEANQVK-biotin), were obtained from Biologica, Co., Nagoya, Japan. Fluorescence labeling of biotinylated Myr47 and its analogs was performed by incubating a biotinylated peptide and Alexa Fluor^®^ 647 streptavidin (Alexa 647; Thermo Fisher Scientific, Waltham, MA, USA) at a molar ratio of 4:1 in phosphate-buffered saline (PBS) at 22 °C for 30 min.

### 2.2. Preparation of BNCs

The preparation of BNCs (i.e., emBNCs were used in this study) and their fluorescence labeling with CellVue Claret (CV) (Molecular Targeting Technologies Inc., West Chester, PA, USA) were previously described [20,21].

### 2.3. Cell Culture

HEK293T, SR-B1-GFP-HEK (i.e., HEK293T cells stably expressing SR-B1-GFP), and GFP-HEK (i.e., those stably expressing GFP) were obtained as previously described [14,15,16]. These cells were maintained in a culture dish in 5% CO_2_ at 37 °C using a culture medium: RPMI1640 medium supplemented with antibiotics and 10% heat-inactivated fetal bovine serum. HepG2 cells were maintained in the same medium using a collagen-coated dish (AGC Techno Glass Co., Ltd., Tokyo, Japan).

### 2.4. Transient Expression of SR-B1-GFP, Mutated SR-B1-GFP, GFP, and NTCP-GFP

The transient expression of SR-B1-GFP, mutated SR-B1-GFP mutants (i.e., S112F, T175A, K151A, K156A, M441A/L448A/L455A, and C-deletion), GFP, and NTCP-GFP in HEK293T cells was performed using PolyMag Neo (OZ Biosciences Inc., San Diego, CA, USA) as described previously [14,15,16]. A plasmid to express NTCP-GFP (catalog number: HG16027-ACG) was purchased from Sino Biological Inc., Beijing, China. Cell lysates, which were obtained from HEK293T cells transfected with or without plasmids for application in SDS-PAGE and Western blot, were prepared as previously described [15,16].

### 2.5. LSM

HEK293T cells stably expressing SR-B1-GFP and GFP (i.e., SR-B1-GFP-HEK and GFP-HEK cells, respectively) were prepared as described previously [14,15,16]. To examine the colocalization of Myr47 and SR-B1-GFP, HEK293T, SR-B1-HEK, and GFP-HEK cells were precultured for 48 h in the RPMI1640 medium (1 × 10^5^/mL, 300 μL/well) in an 8-well chambered coverglass (Thermo Fisher Scientific) that was coated with poly-L-lysine. After culture supernatants were removed, cells were incubated with Alexa 647 (100 nM)-labeled Myr47 (400 nM) in PBS at 22 °C for 30 min. Cells were washed twice with PBS, added ro 200 μL of PBS, and immediately subjected to LSM using FV1000 (Olympus, Tokyo, Japan).

### 2.6. Ligand-Binding Assays Using Flow Cytometry

Plasmid-transfected or untransfected HEK293T cells were collected via trypsinization. Cells were washed twice with PBS at 4 °C via centrifugation. Fluorescence-labeled Myr47 (200 nM) and its derivatives were admixed to cell pellets in tubes. A resultant admixture in a tube was incubated on ice for 1 h, and then cells were washed twice with cold PBS. The fluorescence intensity (FI) of 1 − 3 × 10^4^ cells were analyzed using a flow cytometer (FACSCant™ II; BD Biosciences, Franklin Lakes, NJ, USA). We assessed the binding of fluorescence-labeled ligands, including Myr47, to cells from a geometric mean of FI using software (FlowJo™ 7.6.5; BD Biosciences). The binding of fluorescent ligands to the GFP-positive (GFP^+^) subset of cells was calculated using the following formula: (FI of GFP^+^ subset of cells treated with fluorescence-labeled ligands)—(FI of untreated cells). Data were expressed as means with standard errors in triplicate assays.

### 2.7. Myr47 Uptake Assays Using Flow Cytometry

Myr47 uptake in HEK293T, SR-B1-GFP-HEK, and GFP-HEK cells was examined according to the methods as previously described [15,16,21]. Briefly, cells were cultured in the presence or absence of Alexa 647-labeled Myr47 (final Myr47 concentration of 20 nM) in a 24-well plate for 24 h. After cells were harvested via trypsinization, they were subjected to flow cytometry. The quantification of Myr47 endocytosis was calculated using the following formula: (FI of GFP^+^ subset in Myr47 uptake assays)—(FI of GFP^+^ subset in Myr47 binding assays).

### 2.8. Crosslink of Myr47 and SR-B1 Using BS3

Cells prepared via trypsinization were centrifuged and washed twice with cold PBS. Those (4 × 10^6^) suspended in PBS (0.5 mL) were incubated in the presence or absence of biotinylated Myr47 (7 μM) on ice for 1 h. After being washed twice with cold PBS, cells were treated with or without 10 mM of BS3 (ThermoFisher) on ice for 1 h while being shaken mildly. To stop the reaction of BS3, 0.1 M of glycine was added to the BS3-treated cell suspension, and the resultant mixture was incubated at 22 °C for 5 min. After the cells were washed twice with cold PBS, the preparation of cell lysates using a RIPA buffer and SDS-PAGE was performed as previously described [15,16]. The pull down of proteins crosslinked with biotinylated Myr47 was performed using Dynabeads M-280 streptavidin (streptavidin beads, ThermoFisher). Each cell lysate (100 μL) diluted to 2 mg/mL with distilled water was incubated with streptavidin beads in a microtube at 22 °C for 30 min while rotating, and then these beads were precipitated using a magnet. Precipitants were washed three times with PBS containing 0.05% Tween 40, and then they were treated at 95 °C for 10 min in an SDS-PAGE loading buffer (45 μL) containing 2-mercaptoethanol. An aliquot (10 μL) of a sample was loaded on each lane of a Mini-PROTEAN Precast Gel (Bio-Rad, Hercules, CA, USA) and electrophoresed at 200 V 40 mA for 30 min.

### 2.9. Western Blot

Western blot was principally performed as described previously [14,15,16]. Precision Plus Protein Kaleidoscope Standards (Bio-Rad) were used as molecular weight markers. To detect SR-B1 and NTCP, we employed a rabbit monoclonal anti-human SR-B1 antibody (catalog number: EP1556Y/ab52629; Abcam, Cambridge, UK) and a rabbit polyclonal NTCP (SLC10A1) antibody (catalog number: HPA042727; Sigma-Aldrich, St. Louis, MO, USA) as primary antibodies, respectively, at a dilution of 1:1000. These primary antibodies were reacted to a PVDF filter, to which electrophoresed samples were transferred using an iBlot (ThermoFisher) at 4 °C for 24 h. A horseradish peroxidase (HRP)-conjugated donkey polyclonal antibody against rabbit IgG (catalog number: A16035; ThermoFisher) was used as a secondary antibody at a dilution of 1:2000. An HRP-conjugated mouse anti-GFP antibody (catalog number: 0518-34; Nacalai Tesque, Kyoto, Japan) was used at a dilution of 1:4000–10,000. An HRP-conjugated mouse monoclonal anti-GAPDH antibody (catalog number: 015-25, 473; Fujifilm Wako Pure Chemical Co., Tokyo, Japan) was used at a dilution of 1:10,000. HRP-conjugated antibodies were incubated with samples at 4 °C for 15–30 min. If necessary, we stripped an antibody, which bound the filter once, using a WB stripping solution (Nacalai Tesque). After re-blocking the filter, we performed a subsequent antibody reaction. 

## 3. Results

### 3.1. Binding Properties of SR-B1 and NTCP for Myr47 and Other HBV-Related Ligands

We demonstrated that flow cytometric analysis using a fluorescence-labeled ligand and SR-B1-GFP fusion protein is useful to elucidate the specific binding of a ligand to SR-B1 [14,15,16,21]. Therefore, we applied this method to analysis for the binding of Alexa 647-labeled Myr47 and its analogs to SR-B1-GFP or NTCP-GFP. In addition, we examined CV-labeled BNC binding to SR-B1-GFP and NTCP-GFP and compared the ligand-binding properties of the two receptors.

**Figure 1:**
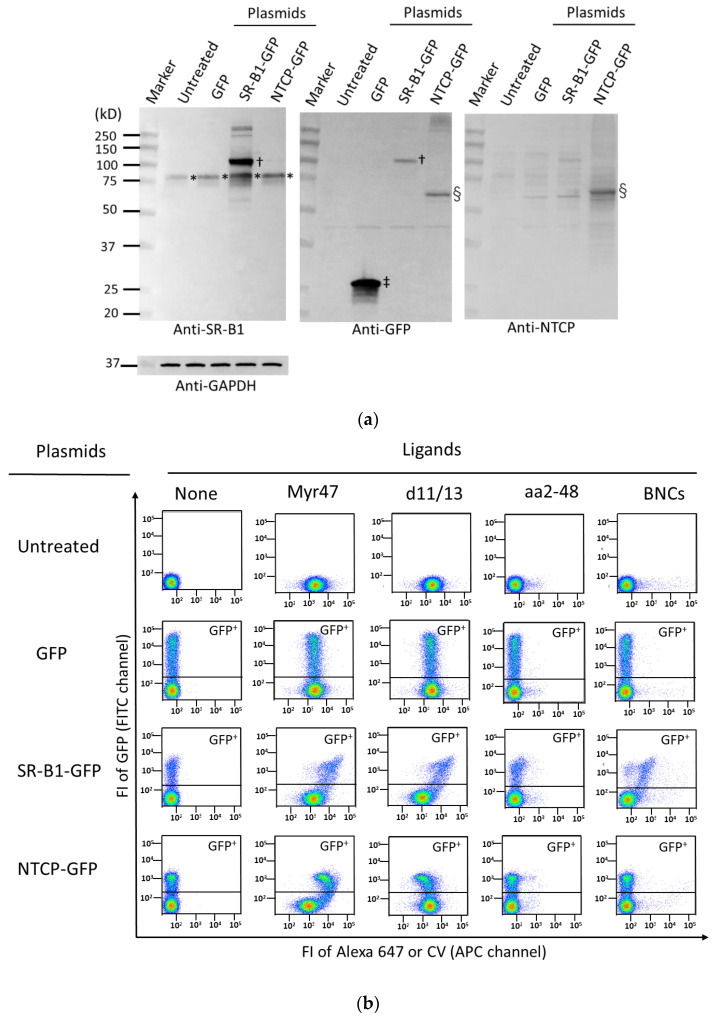

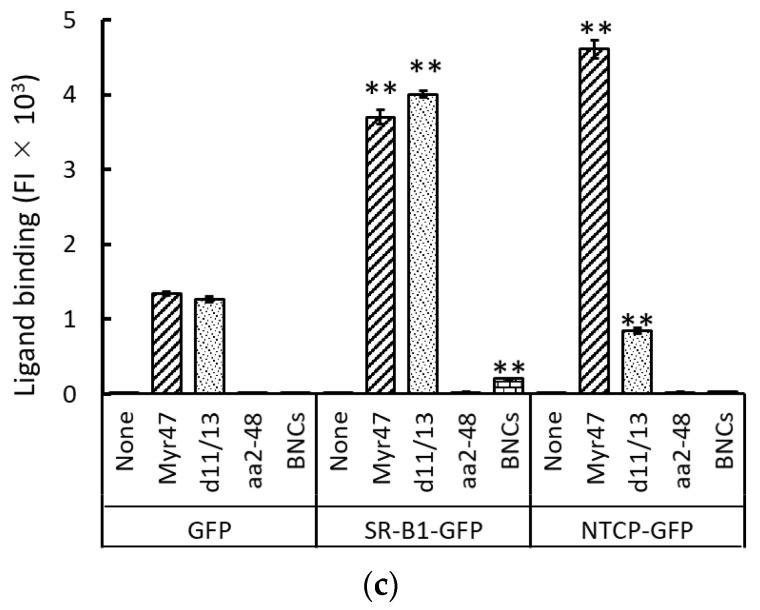
Binding properties of HEK293T cells expressing SR-B1-GFP and NTCP-GFP for Myr47, D-11/13, aa2-48, and BNCs. GFP, SR-B1-GFP, and NTCP-GFP were transiently expressed in HEK293T cells, and the binding of the fluorescence-labeled ligands was examined. (**a**) Western blot to detect GFP, SR-B1-GFP, and NTCP-GFP proteins in HEK293T cells transfected with their expression plasmids. Cell lysates, which were prepared from cells transfected with or without plasmids, were subjected to SDS-PAGE (5 μg protein/lane), and the Western blot analyses were carried out. SR-B1, GFP, NTCP, and GAPDH protein bands were detected using corresponding antibodies, which are indicated in the pictures. The same PVDF filter was repeatedly used for antibody binding after stripping antibodies previously bound to the filter. Symbols *, †, ‡, and § correspond to major bands of SR-B1, SR-B1-GFP, GFP, and NTCP-GFP proteins, respectively. (**b**) Two-parameter dot plot analyses of ligand binding to GFP, SR-B1-GFP, and NTCP-GFP using flow cytometry. HEK293T cells transfected with or without indicated expression plasmids were examined as to whether various fluorescence-labeled ligands bind to them. To obtain these plots, 30,000 cells of each sample were subjected to the analysis. Vertical and horizontal axes of plots show FI of GFP and ligands with flow cytometric channels used for the analyses, respectively. (**c**) Quantified ligand binding of HEK293T cells expressing GFP, SR-B1-GFP, and NTCP-GFP. Ligand binding of the upper gated area (GFP^+^) in each two-parameter dot plot (**a**) was quantified. Data are expressed as means and standard errors (vertical bars) in triplicate assays. To obtain these data, 10,000 cells were analyzed. Statistical analysis was carried out using Student’s *t*-test; ** indicates *p* < 0.01 when two values were compared between the GFP^+^ subsets of cells expressing GFP and SR-B1-GFP or NTCP-GFP.

We confirmed the transient expression of GFP, SR-B1-GFP, and NTCP-GFP in HEK293T cells via Western blot. As shown in Figure 1a, in HEK293T cells transfected with an SR-B1-GFP expression plasmid, SR-B1-GFP protein was detected as a major band near a 100-kD marker by anti-SR-B1 († in the left picture) and anti-GFP († in the middle picture) antibodies. On the other hand, endogenous SR-B1 protein was recognized as about a 75-kD band. In cells expressing GFP, its protein was detected near a 25-kD maker (‡ in the middle picture). In HEK293T cells transfected with an NTCP-GFP expression plasmid, NTCP-GFP protein was found as a band of about 60–70 kD by anti-GFP (§ in the middle picture) and anti-NTCP (§ in the right picture) antibodies.

As shown in the GFP^+^ subset (upper gated area) of two-parameter dot plots (Figure 1b), the binding of Myr47 and D-11/13 to SR-B1-GFP was observed, whereas that of aa2-48 was barely detected. Although Myr47 bound NTCP-GFP, the Myr47 binding pattern differed between SR-B1-GFP and NTCP-GFP. These results suggest that Myr47 binding mode is considerably different between SR-B1-GFP and NTCP-GFP. In contrast to SR-B1-GFP, the expression of NTCP-GFP seemed to reduce the binding of D-11/13. NTCP-GFP, as well as SR-B1-GFP, failed to bind aa2-48, which suggests that the myristoylation of the pre-S1 peptide is important for both Myr47 bindings of SR-B1 and NTCP. BNCs bound SR-B1-GFP, whereas they failed to bind NTCP-GFP, which was consistent with the results of the previous reports [10,21]. Figure 1c shows quantified ligand binding (i.e., FI) to the surfaces of GFP^+^ subsets in cells expressing GFP (control), SR-B1-GFP, and NTCP-GFP. Control cells, which did not express SR-B1-GFP, bound Myr47 and D-11/13. HEK293T cells endogenously express SR-B1. In addition, it is suggested that myristoylated peptides interact with phospholipid membranes in a nonspecific manner [13]. Because of these reasons, it was presumed that Myr47 and D-11/13 bound control cells. SR-B1-GFP-expressing cells bound Myr47 and D-11/13 more than control cells (i.e., 2.8- and 3.2-fold, respectively). These results indicated that SR-B1 can bind both Myr47 and D-11/13. NTCP-GFP-expressing cells also bound Myr47 (i.e., 3.4-fold). However, the D-11/13 binding of these cells was lower than that of the control cells. SR-B1-GFP-expressing cells bound BNCs 9.2-fold more than control cells, whereas BNC binding was not observed in NTCP-GFP-expressing cells. These results indicate that SR-B1 and NTCP have differential binding properties in terms of HBV-related ligands, although both SR-B1 and NTCP receptors can bind Myr47.

### 3.2. Binding of Myr47 to SR-B1 Mutants

**Figure 2:**
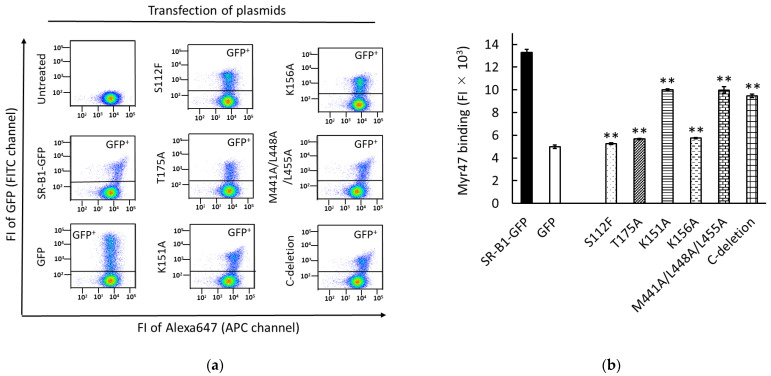
Effects of various mutations in SR-B1 on the binding of Myr47. HEK293T cells transfected with or without indicated plasmids were examined for the binding of Myr47. (**a**) Two-parameter dot plot analyses of Myr47 binding to GFP, SR-B1-GFP, and SR-B1-GFP mutants. HEK293T cells transfected with or without indicated expression plasmids were examined as to whether Myr47 binds to them. To obtain these plots, 30,000 cells of each sample were subjected to the analysis. Vertical and horizontal axes of plots are indicated as FI of GFP and Myr47 with flow cytometric channels used for the analyses, respectively. (**b**) Quantified ligand binding of HEK293T cells expressing GFP, SR-B1-GFP, and SR-B1-GFP mutants. Ligand binding of the upper gated area (GFP^+^) in each two-parameter dot plot (**a**) was quantified. Data are expressed as means and standard errors (vertical bars) in triplicate assays. To obtain these data, 10,000 cells were applied for the analysis. Statistical analysis was carried out using Student’s *t*-test; ** indicates *p* < 0.01 when two values were compared between SR-B1-GFP and mutants.

We previously reported analyses concerning the binding site of BNCs to SR-B1 using HEK293T cells transiently expressing SR-B1-GFP mutants [21]. Western blot data to confirm the expression of these mutants employed here were published in the previous report [16]. To compare between BNC and Myr47 binding to SR-B1, we analyzed Myr47 binding to HEK 293T cells expressing SR-B1-GFP and its mutants. Two-parameter dot plot data and the quantification of Myr47 binding to GFP^+^ subsets in HEK293T cells expressing SR-B1 and its mutants are shown in Figure 2a,b, respectively. S112F and T175A mutations cause the loss of not only the binding of HDL but also that of PC-containing liposomes and BNCs [16,21,22]. We found that these mutations abrogated the binding of Myr47 to SR-B1 too. K156A mutation, which was originally reported to influence silica binding to SR-B1, decreases the binding of PC-containing liposomes and BNCs [16,21,23]. Similarly, this mutation attenuated the binding of Myr47. K151A and M441A/L448A/L455A mutations and the deletion of the C-terminal intracellular domain (i.e., C-deletion) are known to relate SR-B1 multimerization and signal transduction, respectively [24,25]. The two mutations reduce BNC binding to SR-B1, although their reduction rates are lower than the other mutations [16,21]. The same mutations slightly decreased Myr47 binding to SR-B1. As a whole, the effects of mutations in SR-B1 employed here were similar between BNC and Myr47 bindings. These results suggest that the binding sites of SR-B1 for the two ligands are common or substantially close.

### 3.3. Colocalization of Myr47 and SR-B1-GFP in SR-B1-GFP-HEK Cells

**Figure 3:**
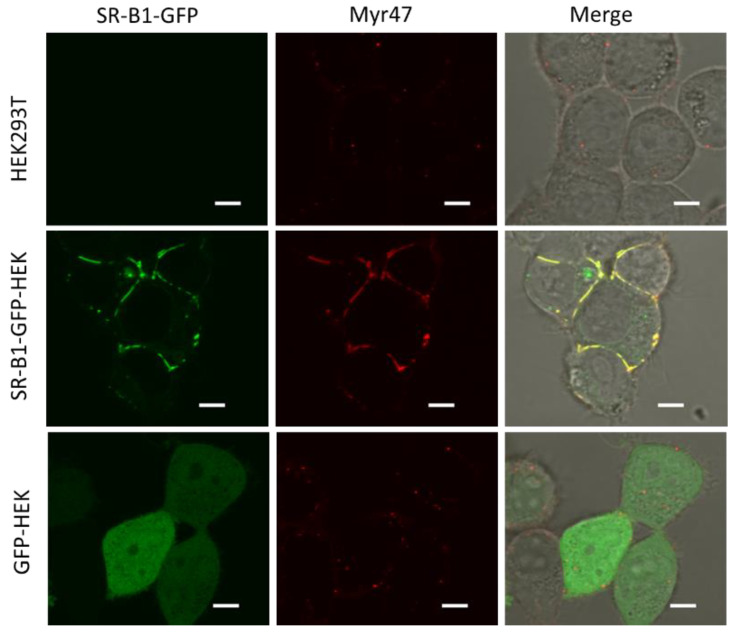
Colocalization of Myr47 and SR-B1-GFP at the surface of SR-B1-GFP-HEK cells. Myr47 binding in HEK293T (upper line), SR-B1-GFP-HEK (middle line), and GFP-HEK cells (bottom line) were observed under LSM. The fluorescence of GFP and Alexa 647-labeled Myr47 is shown in green and red, respectively; Merge: GFP and Myr47 fluorescence and bright filed images are overlaid. Bars indicate 5 μm.

To examine the colocalization of Myr47 and SR-B1-GFP in HEK293T cells, we employed HEK293T cell lines stably expressing SR-B1-GFP (i.e., SR-B1-GFP-HEK cells) or GFP (i.e., GFP-HEK cells). The characteristics of these cell lines have been previously reported [15,16]. As shown in Figure 3, the colocalization of Myr47 and SR-B1-GFP was observed in the surface of SR-B1-GFP-HEK cells under LSM. However, such colocalization was not observed between GFP and Myr47 in GFP-HEK cells. In HEK293T cells that did not express SR-B1-GFP and GFP, dense Myr47 binding, which was observed in SR-B1-GFP-HEK cells, was never detected. These results indicate that Myr47 binds SR-B1-GFP expressed on the cell surface of SR-B1-GFP-HEK cells.

### 3.4. Endocytosis of Myr47 into SR-B1-GFP-HEK Cells

**Figure 4:**
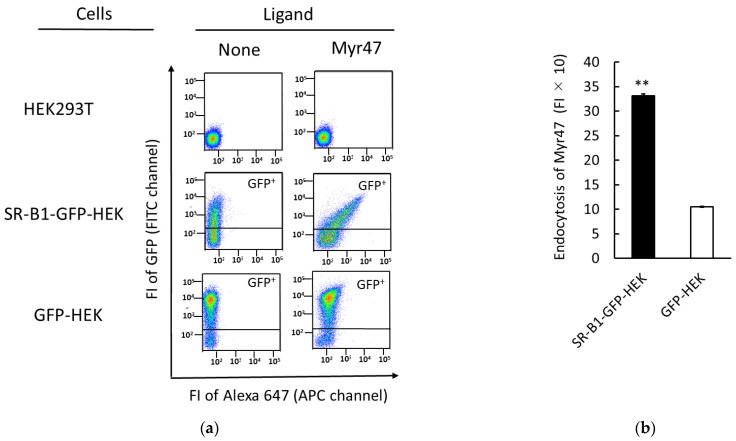
Endocytosis of Myr47 in SR-B1-GFP-HEK cells. Indicated cells were subjected to Myr47 uptake assays. (**a**) Two-parameter dot plot analyses of Myr47 uptake in HEK293T, SR-B1-GFP-HEK, and GFP-HEK cells. To obtain these plots, 30,000 cells of each sample were subjected to the analysis. Vertical and horizontal axes of plots are indicated as FI of GFP and Myr47 with flow cytometric channels used for the assays, respectively. (**b**) Quantified Myr47 endocytosis in SR-B1-GFP-HEK and GFP-HEK cells. Both Myr47 uptake and binding in GFP^+^ subsets in the two-parameter dot plots of SR-B1-GFP and GFP-HEK cells were determined. Quantification of Myr47 endocytosis was carried out by calculating the subtraction of FI of Myr47 bindings from that of Myr47 uptakes. Data are expressed as means and standard errors (vertical bars) in triplicate assays. To obtain these data, 10,000 cells were applied for the analysis. Statistical analysis was carried out using Student’s *t*-test; ** indicates *p* < 0.01.

To elucidate cellular events after Mr47 binding to SR-B1-GFP-HEK cells observed in Figure 3, we examined Myr47 uptake in SR-B1-GFP-HEK cells and compared those in HEK293T and GFP-HEK cells. We applied the methods which we used to assess the uptake of PNPs previously for the determination of Myr47 endocytosis in SR-B1-GFP-HEK cells [15,16,21]. In Myr47 uptake assays, we employed a one-tenth-lower dose of Myr47 than those used in Figure 1 and Figure 2, because the FI values of the uptake assays were far higher than those of the binding assays. As shown in the two-parameter dot plots of Figure 4a, the GFP^+^ subset of SR-B1-GFP-HEK cells incorporated Myr47 more efficiently than those of GFP-HEK or HEK293T cells. Endocytosed Myr47 in the GFP^+^ subsets of SR-B1-GFP-HEK and GFP-HEK cells was quantified by subtracting the FI values of membrane-binding Myr47, which was determined by the same procedure used in Figure 1 and Figure 2, from those of Myr47 uptake assays. Under our experimental conditions, we could not rule out the possibility that the values of membrane-binding Myr47 were underestimated, because Myr47 binding was examined at 4 °C, while the uptake assays were carried out at 37 °C. However, Myr47 bindings to the GFP^+^ subsets of SR-B1-GFP-HEK and GFP-HEK cells were far lower (i.e., about 10 and 5%, respectively) than Myr47 uptakes. Therefore, it was supposed to at least roughly reflect the difference of Myr47 endocytosis between SR-B1-GFP-HEK and GFP-HEK cells. As shown in Figure 4b, endocytosed Myr47 was about three times higher in SR-B1-GFP-HEK cells than in GFP-HEK cells. Considering the results of Figure 3 and Figure 4, it was strongly suggested that Myr47 is endocytosed into SR-B1-GFP-HEK cells at least a part via SR-B1-GFP.

### 3.5. Crosslink of Myr47 and SR-B1-GFP or Endogenous SR-B1 Using BS3

**Figure 5:**
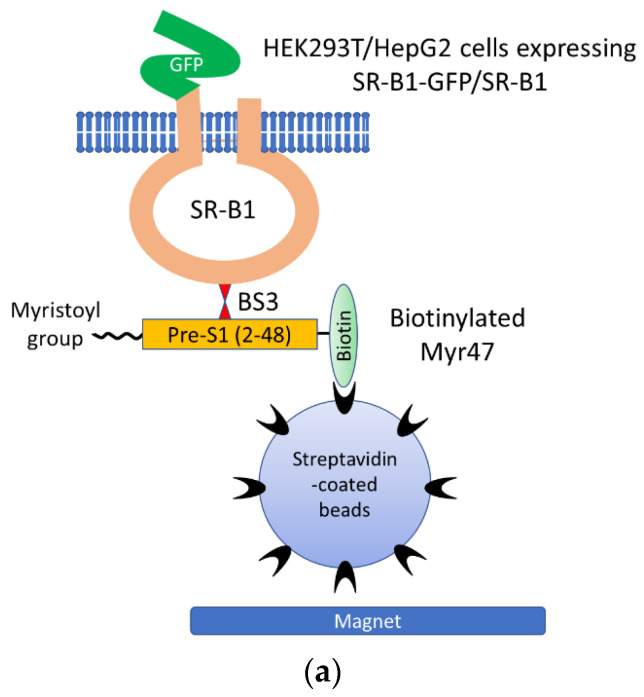

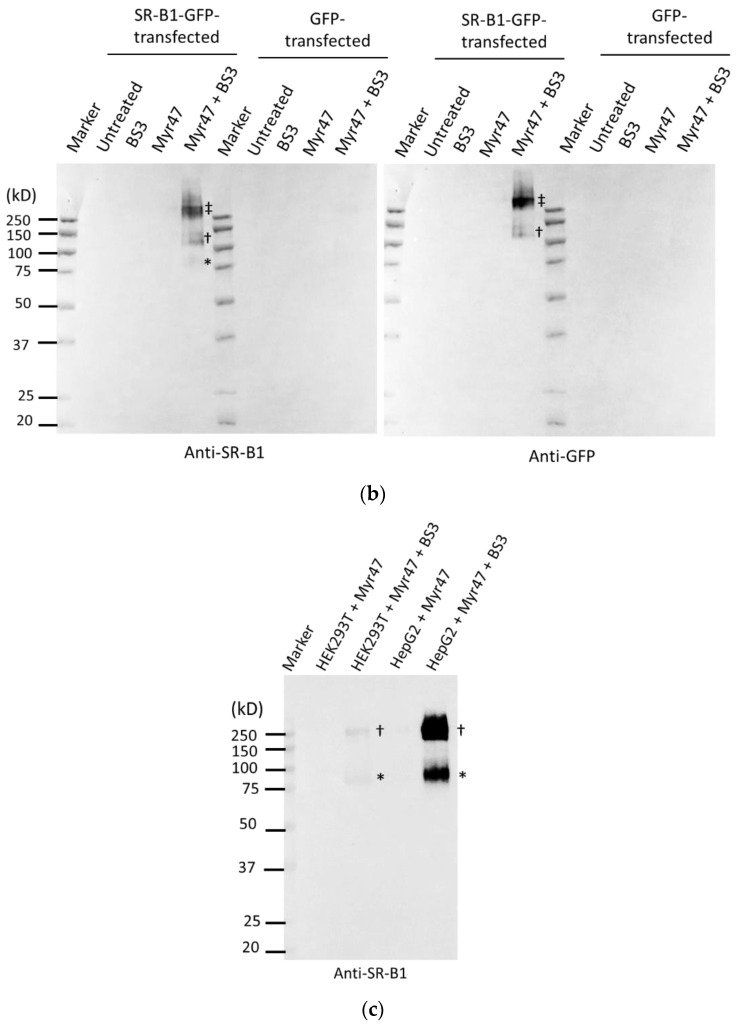
Crosslink of Myr47 and SR-B1-GFP or endogenous SR-B1. (**a**) Illustrated image of pull-down assays to detect SR-B1-GFP or endogenous SR-B1 crosslinked with biotinylated Myr47 using BS3. (**b**) Western blot analyses of pull-down samples prepared from HEK293T cells expressing SR-B1-GFP or GFP after sequential treatment with Myr47 and BS3. HEK293T cells transfected with indicated expression plasmids were incubated with or without biotinylated Myr47. Subsequently, they were treated with or without BS3. Cell lysates were prepared, and then they were applied for pull-down assays using streptavidin-coated beads and a magnet. The same volume (10 μ) of samples was applied for SDS-PAGE and Western blot analyses. Antibodies used for the detection of protein bands are indicated under the picture. Symbols, *, †, ‡, indicate Myr47-binding endogenous SR-B1 multimer, SR-B1-GFP-monomer, and SR-B1-GFP multimer, respectively. (**c**) Western blot analyses of pull-down samples prepared from HEK293T or HepG2 cells expressing endogenous SR-B1 after sequential treatment with Myr47 and BS3. HEK293T or HepG2 cells were treated with biotinylated Myr47 and BS3. Then, pull-down assays and Western blot analyses were carried out as described in (**b**). Symbols, * and †, indicate Myr47-binding endogeous SR-B1 monomer and multimer, respectively.

To confirm the binding of Myr47 and SR-B1, we performed crosslinking of Myr47 and SR-B1 with BS3. Being a water-soluble crosslinker with a spacer arm of 11.4 Å, BS3 is expected to suit analyses for the interaction of exogenous ligands (i.e., Myr47) and cell surface receptors (i.e., SR-B1). In Myr47, the ε amine of the lysine residue at the 37th position from the N terminus can react to the N-hydroxysuccinimide ester of BS3. Our experimental design is illustrated in Figure 5a. HEK293T cells, which were transfected with an expression plasmid of SR-B1-GFP or GFP, and HepG2 cells were incubated in the presence or absence of biotinylated Myr47. After Myr47 was washed out, cells were treated with or without BS3. Then, cells were lysed and cell lysates were applied to streptavidin-coated beads to pull down proteins crosslinked with Myr47. Subsequently, the resultant beads were heat-treated in an SDS-PAGE loading buffer under reducing conditions. An aliquot of samples was subjected to SDS-PAGE, and Western blot analysis was performed. 

As shown in Figure 5b (the left picture), specific bands reacting to anti-SR-B1 antibody were only detected in a sample obtained from SR-B1-GFP-expressing HEK293T cells treated with a combination of Myr47 and BS3. No strong signal was detected in the other samples prepared from SR-B1-GFP-expressing cells treated with or without BS3 or Myr47. Similarly, specific bands were only detected by anti-GFP antibody in the sample of SR-B1-GFP-expressing HEK293T cells treated with both Myr47 and BS3 (the right picture). No signal was detected by either anti-SR-B1 antibody or anti-GFP antibody in any samples prepared from GFP-expressing HEK293T cells under the conditions employed here. In a sample obtained from SR-B1-GFP-expressing HEK293T cells treated with Myr47 and BS3, three bands were mainly detected, as indicated with symbols in the left picture. A faint band (*) was detected by the anti-SR-B1 antibody near a 75-kD maker. However, a corresponding band was not detected by the anti-GFP antibody. Based on the molecular size and lack of GFP antigenicity, this band might be derived from endogenous SR-B1. The other two bands (indicated with † and ‡) were detected at 100–150 kD and more than 250 kD, respectively, by both antibodies. Bands (†) detected at 100–150 kD appeared to be derived from an SR-B1-GFP monomer binding Myr47, based on molecular size. As these were not a single band, the number of Myr47 molecules binding to SR-B1-GFP would be heterogeneous. Unexpectedly, the strongest signal was detected in the bands of more than 250 kDa. Based on molecular size, these bands were presumed to be a complex of SR-B1 multimer and Myr47. These results indicate that Myr47 binds SR-B1. Furthermore, it is suggested that Myr47 preferentially binds SR-B1 multimer rather than monomer in HEK293T cells.

As HEK293T cells are a cell line established from human embryonic kidney cells, we next compared Myr47 binding to endogenous SR-B1 in HEK293T and HepG2 cells, a hepatic cell line. Myr47-linked endogenous SR-B1 was detected in the pull-down assays as faint bands at 75–100 kD and more than 250 kD in HEK293T cells (indicated with * and † in Figure 5c, respectively); it should be noted that a very faint band at more than 250 kD was observed in the lane of GFP-expressing HEK293T cells treated with Myr47 and BS3 in the left Western blot of Figure 5b. In Figure 5c, bands with stronger signals were found in HepG2 cells at the same positions in HEK293T cells. The reason that strong signals were obtained in the assays using HepG2 would be due to the more abundant expression of endogenous SR-B1 in HepG2 cells than in HEK293T cells. In addition, a band at more than 250 kD exhibited a greater signal than that at 75–100 kD in HepG2 cells. These results indicate that Myr47 binds both SR-B1 monomer and multimer not only in HEK293T cells but also in HepG2 cells.

## 4. Discussion

To clarify the interaction between a ligand and a cell membrane receptor, flow cytometric analysis using a fluorescence-labeled ligand and cells expressing receptor-GFP fusion protein is a potent approach. By applying this method, we demonstrated that SR-B1 plays a pivotal role in the binding and endocytosis of liposomes containing PC and PE in HEK293T cells [12,13]. Based on these results, we presumed that SR-B1 would play a role in the cellular recognition of a variety of PNPs. We investigated whether BNCs can bind SR-B1 and then found that SR-B1 functions as a receptor for BNCs [21]. On the other hand, an N-terminal myristoylated pre-S1 peptide (i.e., Myr47) was demonstrated to inhibit both HBV and HDV infection to hepatocytes [4,5,6,7,8]. As we considered that SR-B1 would be able to bind not only BNCs but also Myr47, we examined the binding of Myr47 to SR-B1. In this study, we demonstrated that Myr47 binds SR-B1, based on the results of (i) flow cytometric analyses using fluorescence-labeled Myr47 and HEK293T cells expressing SR-B1-GFP, (ii) the colocalization of fluorescence-labeled Myr47 and SR-B1-GFP in SR-B1-GFP-HEK cells, and (iii) the crosslinking of Myr47 and SR-B1-GFP using BS3. We used HEK293T cells to demonstrate the interaction of Myr47 and SR-B1-GFP. This cell line is widely used in virology and cellular biology experiments because it has simian virus 40 (SV40) large T antigen, which can promote the replication of transfected DNA plasmids containing SV40 Ori [26]. Therefore, we mainly used HEK293T cells in transient expression experiments in this study.

We compared ligand-binding properties between SR-B1 and NTCP. Fluorescence-labeled Myr47 bound both SR-B1-GFP and NTCP-GFP expressed on HEK293T cells. However, the Myr47-binding patterns of the two-parameter dot plots were different between the two receptors. Roughly, Myr47 binding to SR-B1-GFP seemed to correlate between the amount of Myr47 bound and the expression level of SR-B1-GFP in HEK293T cells (i.e., cells expressing SR-B1-GFP at a high level bind Myr47 more than those at a low level). On the other hand, Mry47 binding to NTCP-GFP appeared to be limited at a certain level (i.e., cells expressing NTCP-GFP at a high level do not always bind Myr47 more than those at a low level), although it remains unclear which factor caused the difference in Myr47 binding between the two receptors. Myr47 has been shown to have the inhibitory activity of HBV infection to hepatic cells, whereas D-11/13 and aa2-48 do not have such activity [7,8]. The ligand-binding properties of NTCP-GFP matched well with the reported HBV-infection-inhibitory activity of these peptides. However, SR-B1-GFP bound D-11/13 as well as Myr47. In NTCP-GFP-expressing HEK293T cells, D-11/13 binding seemed to be rather reduced, although the reason for this was unclear. In addition, BNCs bound SR-B1-GFP, but they did not NTCP-GFP. These results demonstrated that SR-B1 and NTCP have differential binding properties regarding HBV-related ligands.

Here, we showed that SR-B1-GFP-HEK cells endocytosed Myr47 more efficiently than GFP-HEK and HEK293T cells. As we demonstrated that Myr47 can bind SR-B1 by various approaches in this study, our results strongly suggest that the binding of Myr47 to SR-B1 induces its endocytosis.

In the previous report, we demonstrated that SR-B1 can bind BNCs [21]. In this study, we showed that SR-B1 binds Myr47. In addition, analyses of Myr47 binding sites using SR-B1 mutants suggested that both binding sites of BNCs and Myr47 are similar or close. Considering these results, it is strongly suggested that SR-B1 is involved in the cellular binding of HBV. SR-B1 mRNA is abundantly expressed in the liver [27]. Using pull-down assays, we showed that Myr47 bound endogenous SR-B1 in HepG2 cells. NTCP-expressing HepG2 cells are frequently used for HBV infection experiments [10]. Therefore, there is a possibility that SR-B1 plays a role in the binding of HBV in hepatic cells. In some viruses including hepatitis C virus, SR-B1 is known to participate in viral binding to cells and infectious processes with various other receptors [28]. However, no evidence has been reported so far that SR-B1 is related to HBV infectivity. Therefore, even if SR-B1 is involved in HBV cellular binding, it might have little influence on the establishment of HBV infection. Anyway, a precise evaluation of SR-B1 will be necessary to determine whether it is related to the process of HBV infection. If SR-B1 does not have any effects on HBV infectivity, examining the binding of Mry47 derivatives to SR-B1 might be helpful to design and select new inhibitors, which can bind NTCP more specifically than Myr47. 

By the present study using a crosslinker (BS3) and pull-down assay, it was suggested that Myr47 bound SR-B1 multimer rather than monomer. SR-B1 has been reported to form a multimer on plasma membranes [24]. In addition, it has been reported that SR-B1 multimer functions in ApoA-I binding and cholesterol efflux, whereas its monomer does not [29]. Therefore, our findings, the preferential binding of Myr47 to SR-B1 multimer, suggest that Myr47 can bind functional SR-B1 multimer. Our approach employed here will be useful in revealing not only the binding nature of SR-B1 but also that of NTCP to Myr47.

## Data Availability

All data generated and analyzed during this study are included in this article.
